# Effects of a selective 5-HT4 agonist and an alpha-2 adrenoceptor antagonist on etorphine-induced impairment across the oxygen cascade in immobilised sheep (*Ovis aries*): a randomised, prospective, and controlled trial

**DOI:** 10.1186/s12917-026-05790-4

**Published:** 2026-08-07

**Authors:** Hathaipat Rattanathanya, Anna Binetti, Friederike Pohlin, Susana C.M. Ferreira, Christina Braun, Johannes Schramel, Leith C.R. Meyer, Anna Haw, Marja Raekallio, Stefan Böhmdorfer, Szilvia Kalogeropoulu, Martina Mosing, Gabrielle Stalder

**Affiliations:** 1 Research Institute of Wildlife Ecology, Department of Interdisciplinary Life Sciences, University of Veterinary Medicine Vienna, Vienna, Austria; 2 Chulabhorn Royal Academy, Bangkok, Thailand; 3 Anaesthesiology and Intensive Care, Clinical Centre for Small Animal Health and Research, Clinical Department for Small Animals and Horses, University of Veterinary Medicine Vienna, Vienna, Austria; 4 Department of Production Animal Studies and Centre for Veterinary Wildlife Research, Faculty of Veterinary Science, University of Pretoria, Pretoria, South Africa; 5 Animal Welfare Science and Bioethics Centre, School of Veterinary Science, Massey University, Palmerston North, New Zealand; 6 Brain Function Research Group, Department of Physiology, School of Biomedical Sciences, University of the Witwatersrand, Johannesburg, South Africa; 7 Faculty of Veterinary Medicine, Department of Equine and Small Animal Medicine, University of Helsinki, Helsinki, Finland; 8 Institute of Chemistry of Renewable Resources, BOKU University, Vienna, Austria

**Keywords:** BIMU-8, Etorphine, Hypoxaemia, Opioid, Vatinoxan

## Abstract

**Background:**

Etorphine, a highly potent opioid, disrupts multiple steps of the oxygen cascade, including alveolar ventilation, pulmonary gas exchange, systemic oxygen transport, and tissue-level oxygen utilisation, resulting in hypoxaemia and cellular hypoxia during immobilisation of wild ungulates. To evaluate potential mitigating agents, a randomised, prospective, controlled crossover design was used in etorphine-immobilised sheep (0.05 mg·kg^−1^, intramuscularly, *n* = 6). After etorphine administration, animals received either the serotonergic agonist BIMU-8 (1.5 mg·kg^−1^), the alpha-2 adrenoceptor antagonist vatinoxan (0.15 mg·kg^−1^), or sterile water (control) intravenously with a four-week washout period. Immobilisation was reversed with naltrexone 19 min after treatment administration. Sequential steps of the oxygen cascade were assessed using thoracic electrical impedance-derived variables (respiratory rate (RR_EIT_) and tidal impedance variation (TIV)) for ventilation, blood gas analysis and venous admixture for pulmonary gas exchange, oxygen delivery (DO_2_) for oxygen transport, and oxygen extraction ratio (ER) for tissue oxygen utilisation. Measurements were obtained at baseline, after etorphine administration, at five-minute intervals following treatment, and after naltrexone. Data was analysed using linear mixed-effects models.

**Results:**

Etorphine induced respiratory depression, characterised by reduced RR_EIT_ (*p* < 0.001) and TIV (*p* = 0.01), and impaired gas exchange, indicated by increased venous admixture (*p* < 0.001) compared with baseline, causing hypercapnia and hypoxaemia in all treatments. Neither BIMU-8 nor vatinoxan improved these variables compared with the control. Consequently, progressive decreases in arterial partial pressure of oxygen (F_3,90_ = 104.82, *p* < 0.001) were observed across treatments during immobilisation, resulting in persistently reduced DO_2_ (F_3,84_ = 51.55, *p* < 0.001) and increased ER (F_3,90_ = 8.09, *p* < 0.001) at similar levels in all treatments. All variables normalised after naltrexone administration.

**Conclusions:**

Etorphine profoundly impaired multiple sequential steps of the oxygen cascade in sheep, resulting in hypoxaemia, reduced DO_2_, and compensatory increases in ER. BIMU-8 and vatinoxan did not effectively mitigate these disturbances at any level of the cascade compared with the control. Future research is needed to develop targeted strategies that address the key mechanisms underlying the disruptions in the oxygen cascade during etorphine immobilisation.

**Supplementary Information:**

The online version contains supplementary material available at https://doi.org/10.1186/s12917-026-05790-4.

## Background

Chemical immobilisation, particularly with highly potent opioids such as etorphine, is essential for the conservation, management, and veterinary care of large wild herbivores [1–3]. Although etorphine is widely used in ungulates due to its high potency and ability to provide rapid and reliable immobilisation in species such as rhinoceros, bison, moose, and antelope, respiratory compromise remains a major concern [4–7]. Evidence indicates that etorphine disrupts multiple sequential steps of the oxygen cascade. By directly activating mu-opioid receptors within the pre-Bötzinger complex (PBC), etorphine induces hypoventilation, characterised by decreased minute ventilation and subsequent hypercapnia [8–10]. Etorphine also induces pulmonary hypertension, alters fluid dynamics, and causes ventilation–perfusion mismatch, thereby impairing pulmonary gas exchange and increasing venous admixture [11, 12], likely via sympathomimetic-mediated pulmonary and systemic vasoconstriction [12–14]. Consistent with limited ventilation and gas exchange, hypoxaemia (arterial partial pressure of oxygen (PaO_2_) < 60 mmHg) is consistently reported in etorphine-immobilised animals [4–7]. If untreated, hypoxaemia, especially when combined with reduced cardiac output (CO), can result in insufficient tissue oxygen delivery (DO_2_), leading to tissue hypoxia and potentially lethal effects. Furthermore, etorphine immobilisation is known to increase oxygen requirements due to elevated muscle activity, such as tremors, leading to increased oxygen consumption (VO_2_) and a higher oxygen extraction ratio (ER) when DO_2_ is reduced [14]. To mitigate etorphine-induced adverse effects, two pharmacological agents show considerable potential, namely BIMU-8 and vatinoxan. BIMU-8, a selective 5-hydroxytryptamine 4 (5-HT4) receptor agonist, may alleviate the disadvantages of etorphine by enhancing alveolar ventilation and pulmonary gas exchange. Together with mu-opioid receptors, 5-HT4 receptors are co-expressed on respiratory neurons in the PBC. However, stimulation of 5-HT4 receptors results in increased respiratory drive and minute ventilation [15, 16]. Additionally, BIMU-8 has been shown to mitigate pulmonary hypertension and improve gas exchange in etorphine-immobilised sheep and goats [17, 18]. Although the exact pathway remains unclear, 5-HT4 receptors have been identified in the pulmonary veins of sheep, and their activation leads to vasodilation [19]. Among available 5-HT4 receptor agonists, BIMU-8 has shown the most promising effect [20]. For example, zacopride alleviated respiratory depression in etorphine-immobilised goats. However, it appeared to interfere with hypotonic immobility [21]. Thus, BIMU-8 was selected for this study due to its potential beneficial effects on ventilation and gas exchange, and its possible contribution to mitigating etorphine-induced hypoxaemia and hypoxia without compromising immobilisation quality.

Vatinoxan is a peripheral alpha-2 adrenoceptor antagonist, acting mainly outside the blood-brain barrier. It has been shown to ameliorate pulmonary hypertension [22–24] and promote cardiovascular function without reversing the desired sedative effects in a range of wild and domestic animals administered alpha-2 adrenoceptor agonists [25–27]. Antagonising the effects of endogenous catecholamines on alpha-2 adrenoceptors in the pulmonary vasculature with vatinoxan may attenuate etorphine-induced sympathetic-mediated vasoconstriction, thereby reducing pulmonary hypertension. Even though vatinoxan did not significantly reduce pulmonary arterial pressures in etorphine-immobilised sheep, as previously reported, some degree of peripheral vasodilation was suggested [18]. Moreover, vatinoxan’s ability to improve cardiovascular function, particularly through increasing CO, suggests that it may also enhance DO_2_. This is expected, as DO_2_ is determined by arterial oxygen content (CaO_2_) and CO [28]. By improving pulmonary perfusion and increasing CO, vatinoxan may facilitate more efficient gas exchange and increase DO_2_ in etorphine-immobilised animals.

**Fig. 1:**
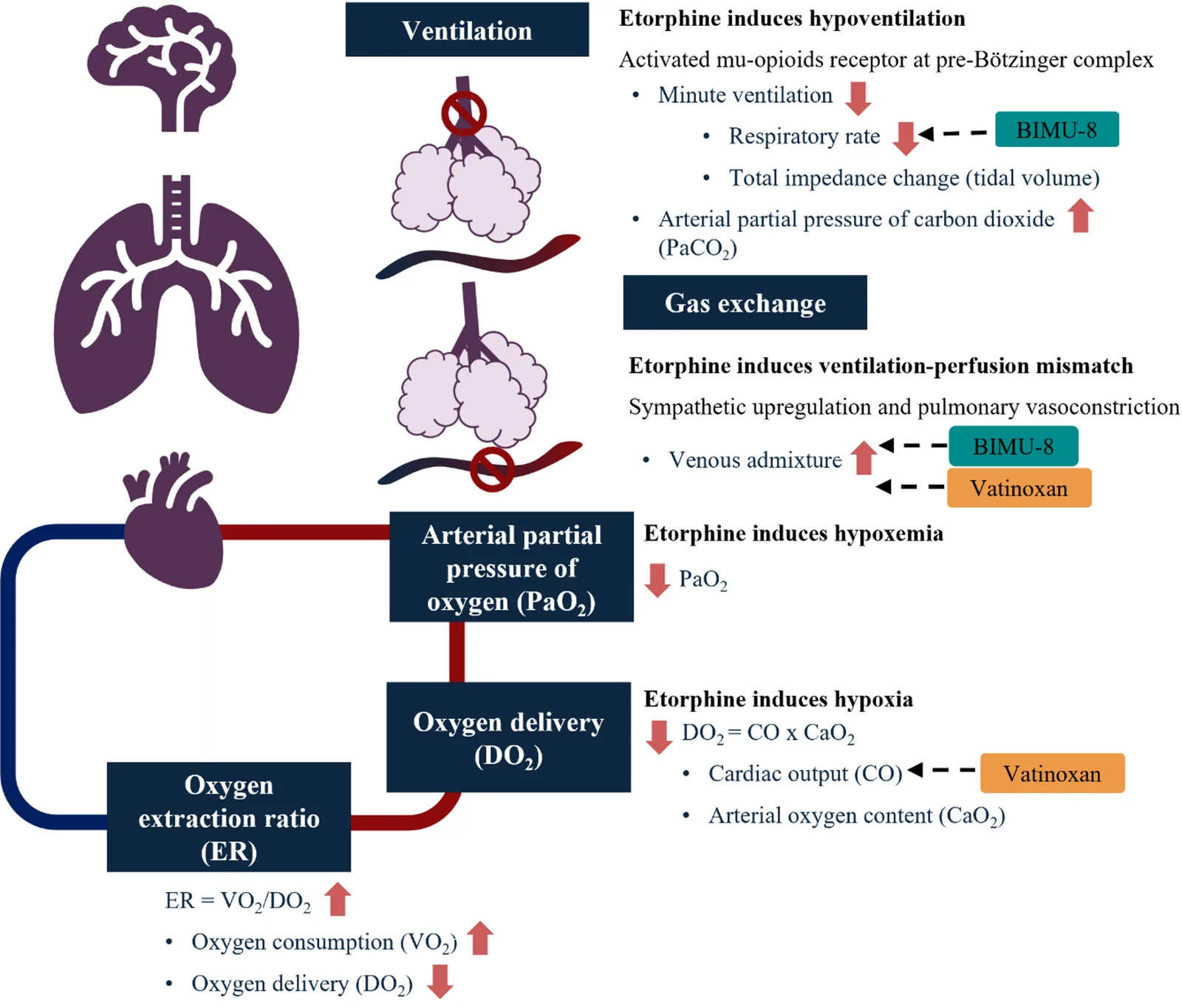
Reported effects of etorphine on the oxygen cascade and the hypotheses tested in this study. Etorphine impairs ventilation and gas exchange, causing hypoxaemia and hypoxia. BIMU-8 and vatinoxan were evaluated in this study for their potential to counteract etorphine-induced respiratory compromise at different levels of the oxygen cascade

The aim of this study was to systematically evaluate the integrity of the entire oxygen cascade during etorphine immobilisation, from alveolar ventilation and pulmonary gas exchange to convective oxygen transport and peripheral oxygen extraction at the tissue level, and to determine whether the novel drugs, BIMU-8 and vatinoxan, could counteract etorphine-induced adverse effects, specifically on alveolar ventilation, gas exchange, DO_2_, and ER in the peripheral tissues. Sheep were selected as a model for wild ungulates due to their physiological similarity and previous reports indicating that responses to etorphine, such as hypoventilation, pulmonary hypertension, and hypoxaemia, are similar to those observed in wild ungulates [29]. In addition, the use of sheep allows animals to be trained to become comfortable with handling and to remain calm in a consistent position, while also providing the opportunity to obtain baseline and recovery measurements that are generally not feasible in wildlife. We hypothesised that etorphine would impair multiple sequential steps of the oxygen cascade, resulting in reduced DO_2_ and increased ER; and that (1) administration of BIMU-8 would improve ventilation by increasing respiratory drive and minute ventilation and pulmonary gas exchange by reducing pulmonary hypertension, whereas (2) vatinoxan would enhance pulmonary gas exchange by reducing sympathetically-mediated pulmonary hypertension and systemic oxygen transport by improving cardiovascular funtion (Fig. 1). Consequently, both agents were expected to mitigate etorphine-induced hypoxaemia and hypoxia.

## Materials and methods

### Study design

A randomised, controlled, prospective crossover trial was conducted using six healthy adult female Tyrolean Mountain sheep (*Ovis aries*; age 5.9 ± 0.3 years). Every animal came from the the VetFarm of the University of Veterinary Medicine, Vienna, Austria, and was owned by the institution. Based on prior studies and an a priori power analysis, a sample size of six sheep was considered sufficient to detect a 10 mmHg difference in mean PaO_2_, assuming normally distributed residuals, a standard deviation of 4, an alpha level of 0.05, and a statistical power of 0.95. Each sheep was immobilised with etorphine (0.05 mg·kg^−1^ Captivon, Wildlife Pharmaceuticals, White River, South Africa) administered into the *quadriceps femoris* muscle and, seven minutes later, randomly assigned (randomizer.org) to receive one of three treatments administered intravenously into the jugular vein, by a non-blinded investigator: BIMU-8 (1.5 mg·kg^−1^; magistral preparation BOKU, Vienna, Austria) [30], vatinoxan HCl (0.15 mg·kg^−1^; Vetcare Ltd., Salo, Finland), or sterile water (control) at an equivalent volume of 0.08 mg·kg^−1^. This dose of etorphine was selected based on a previous report demonstrating that it induces cardiorespiratory compromise in sheep [29]. In the present study, the dose of BIMU-8 was determined in a pilot study. Although an intravenous dose of 3 mg·kg^−1^ had previously shown beneficial effects in etorphine-immobilised goats [17], it induced tachyarrhythmias in sheep and was therefore reduced by half to 1.5 mg·kg^−1^. In contrast, the dose of vatinoxan was based on established use in sheep, where it has been shown to improve cardiopulmonary function during administration of an alpha-2 adrenoceptor agonist [31].

Naltrexone (1 mg·kg^−1^; 20 mg per mg etorphine, Trexonil, Wildlife Pharmaceuticals Pty Ltd., White River, South Africa) was administered intravenously 19 min after treatment administration. Each sheep received each treatment with a washout period of four weeks between trials. Upon completion of the experimental procedures, animals underwent veterinary assessment and were then allocated to private care via the institution’s approved rehoming programme. No animals were euthanised as part of this study.

To simulate conditions in wild ungulates and evaluate the effects of the treatments, etorphine-immobilised sheep breathed spontaneously on room air without supplemental oxygen or ventilatory support. Relevant outcome variables (Table 1) were measured or calculated using data from electrical impedance tomography (EIT), arterial and mixed venous blood analyses, and cardiac output monitoring.

**Table 1:** Measurement and calculation of the outcome variables representing the oxygen cascade. (a) tHb = total haemoglobin concentration measured by blood gas analyser (g·dL^−1^); (b) PB = barometric pressure measured by blood gas analyser from corresponding time point (mmHg); (c) PH_2_O = partial pressure of water vapour (= 47 mmHg); (d) FiO_2_= fraction of inspired oxygen (= 0.209); (e) RQ = respiratory quotient in ruminants (= 0.99) [32]; (f) SaO_2_ = arterial oxygen saturation (%); (g) SvO_2_ = mixed venous oxygen saturation (%); and (h) PvO_2_ = mixed venous partial pressure of oxygen (mmHg)

	Outcome Variables	Measurement or calculation
*Ventilation*	1) Respiratory rate (RR_EIT_)	EIT: Number of end-inspirations per minute
	2) Tidal impedance variation (TIV)	EIT: Total Impedance change (ΔZ) between start and end of each inspiration, as a surrogate for tidal volume
	3) Minute tidal impedance variation (TIV_MIN_)	EIT: TIV_MIN_ = TIV x RR_EIT_, as a surrogate for minute ventilation
	4) Arterial partial pressure of carbon dioxide (PaCO_2_)	Arterial blood gas analysis
*Gas exchange*	Venous admixture (Qs/Qt)	Arterial and mixed venous blood gas analysis $\mathrm{Qs/Qt}=\left(\mathrm{CcO}_2-\mathrm{CaO}_2\right)/\left(\mathrm{CcO}_2-\mathrm{CvO}_2\right)$ $$\begin{aligned}\mathrm{CcO}_2&=\text{Capillary Oxygen Content}\left(\mathrm{mL/dL}\right)\\&=\left(1.34 \times\mathrm{tHb}^\mathrm{a}\right)+\left(0.0031 \times\mathrm{PAO}_2\right)\end{aligned}$$ $$\begin{aligned}\mathrm{PAO}_2&=\text{Alveolar Oxygen partial pressure}\\&=\left(\mathrm{PB}^\mathrm{b}-\mathrm{PH}_2\mathrm{O}^\mathrm{c}\right)\times\mathrm{FiO}_{2}{^\mathrm{d}}-\left(\mathrm{PaCO}_2/\mathrm{RQ}^\mathrm{e}\right)\end{aligned}$$ $$\begin{aligned}\mathrm{CaO}_2&=\text{Arterial Oxygen Content} \left(\mathrm{mL/dL}\right)\\&=\left(\mathrm{tHb}\times1.34 \times\mathrm{SaO}_2{^\mathrm{f}}\right)+\left(\mathrm{PaO}_2\times0.003\right)\end{aligned}$$ $$\begin{aligned}\mathrm{CvO}_2&=\text{Mixed Venous Oxygen Content }\left(\mathrm{mL/dL}\right)\\&=\left(\mathrm{tHb}\times1.34 \times\mathrm{SvO}_2{^\mathrm{g}}\right)+\left(\mathrm{PvO}_2{^\mathrm{h}}\times0.003\right)\end{aligned}$$
*Arterial oxygen*	Arterial partial pressure of oxygen (PaO_2_)	Arterial blood gas analysis
*Oxygen delivery*	Oxygen delivery (DO_2_)	$\mathrm{DO}_2\left(\mathrm{mL/min}\right)=\text{cardiac output}\left(\mathrm{CO};\mathrm{L/min}\right)\times\mathrm{CaO}_2\times10$
*Oxygen extraction*	Oxygen extraction ratio (ER)	$\mathrm{ER}=\mathrm{VO}_2/\mathrm{DO}_2$ $$\begin{aligned}\mathrm{VO}_2&=\text{Oxygen consumption}\left(\mathrm{mL/min}\right)\\&=\mathrm{CO}\times\left(\mathrm{C}\left(\mathrm{a-v}\right)\mathrm{O}_2\right)\times10\end{aligned}$$ $\mathrm{C}\left(\mathrm{a-v}\right) \mathrm{O}_2\left(\mathrm{mL/dL}\right)=\mathrm{CaO}_2-\mathrm{CvO}_2$

**Fig. 2:**
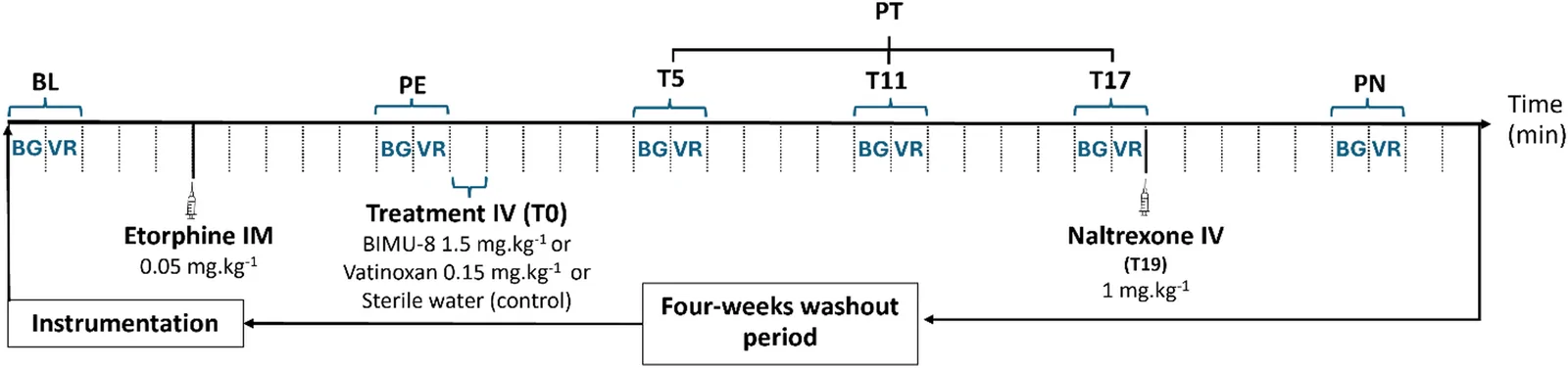
Experimental timeline. Each sheep (*n* = 6) received three treatments; BIMU-8, vatinoxan, or sterile water (control), in a randomised, controlled, crossover trial with a four-week washout period between treatments. The experimental procedure was divided into four phases in which data were collected: baseline (BL), post-etorphine (PE), post-treatment (PT), and post-naltrexone (PN). Animals were instrumented before BL. At each time point within these phases, arterial and mixed venous blood samples (BG) were collected first, followed by a one-minute recording of CO measurements and EIT variables (VR). Etorphine IM: administration of 0.05 mg·kg^−1^ etorphine hydrochloride IM, Post etorphine (PE): measurements after etorphine administration, Treatment IV (T0): administration of 1.5 mg·kg^−1^ BIMU-8, 0.15 mg·kg^−1^ vatinoxan or sterile water equal in volume to the amount of other treatments in mL (control) IV over one minute, Time point T5, T11, T17: measurements after treatments (PT), Naltrexone IV (T19): administration of 1 mg·kg^−1^ naltrexone, Post naltrexone (PN) : measurements after 5 min of naltrexone administration

Further details regarding experimental setup, animal model and handling, methodology and exclusion criteria have been described in detail in Rattanathanya et al. 2026 [18]. For clarity and ease of understanding, the overall experimental timeline (Fig. 2) and relevant descriptions of the experimental setup are nevertheless included in this manuscript.

### Instrumentation

Respiratory rate (RR_EIT_) and tidal impedance variation (TIV), derived from total lung impedance as surrogate for tidal volume, were assessed using EIT. The sheep were clipped circumferentially around the central thorax, specifically at the 5th − 6th intercostal space for application of a customised, stretchable EIT belt. Non-conductive ultrasound gel was applied to the clipped thoracic area, and the EIT belt, containing 32 electrodes, was placed over the gel. The belt was connected to the EIT device, BB2/BBVet (Sentec, Landquart, Switzerland), and after confirming adequate electrical contact between the skin and electrodes, providing stable signal readings, a bandage was wrapped over the belt to guarantee electrode contact to skin and prevent drying of the gel. After EIT placement, the sheep was positioned in a sling supporting a standing posture, with only the tips of the feet in contact with the ground and the head held upright throughout the experiment.

For arterial and mixed venous blood sampling, arterial and pulmonary arterial catheters were placed. The ears and left neck were aseptically prepared and locally anaesthetised with lidocaine (Xylanaest purum 2%, Gebro Pharma GmbH, Fieberbrunn, Austria), 0.1 mL intradermally over the auricular artery and 2 mL subcutaneously at the mid-neck to desensitise the skin and underlying tissues. An 18-gauge IV catheter (Vasofix Safety, B. Braun Melsungen AG, Melsungen, Germany) was placed into one of the auricular arteries. Simutaneously, the skin over the left jugular vein was incised to insert a percutaneous sheath introducer set (8.5 Fr., 10 cm sheath; Teleflex, Arrow International LLC, Morrisville, NC, USA). A continuous cardiac output thermodilution pulmonary arterial catheter (Swan-Ganz Catheter, Edwards Lifesciences, Irvine, CA, USA) was then advanced through the vein via the introducer into the right ventricle and subsequently into the pulmonary artery under pressure guidance. The catheter tip was positioned in the pulmonary artery, and mixed venous blood was collected from its distal port. Both the arterial and pulmonary arterial catheters were connected to pressure transducers mounted at the level of the heart for monitoring of arterial and pulmonary arterial pressures as part of the cardiovascular study [18]. The pulmonary arterial catheter was additionally connected to a Vigilance II continuous cardiac output monitor (Edwards Lifesciences, Irvine, CA, USA) for measurement of cardiac output and body temperature.

### Data collection

The experimental timeline and data collection time points are shown in Fig. 2. Variables derived from EIT, arterial and mixed venous blood samples, and cardiac output were collected 5 min before and after etorphine injection (BL and PE, respectively), at three time points at 5-minute intervals following treatment (PT), and at a final time point 5 min after naltrexone administration (PN).

Variables derived from EIT, including RR_EIT_ and TIV, were recorded continuously using the EIT device throughout the experiment. The EIT electrodes were driven in a skip-4 pattern, and data were collected at a frame rate of 47 images per second. EIT data were analysed using ibex software (Sentec, Landquart, Switzerland), and TIV was reported in arbitrary units (AU).

One minute prior to arterial and mixed venous blood sampling, 5 mL of blood were withdrawn and discarded from the dead space of each catheter. Subsequently, 3 mL of arterial and mixed venous blood were collected into heparinised blood gas syringes (BD Vacutainer, Becton Dickinson Company, Franklin Lakes, NJ, USA). Both catheters were flushed with sterile 0.9% sodium chloride solution following sample collection. Within 5–10 min of collection, the samples were analysed using an automated blood gas analyser (Cobas b 123 POC system, Roche Diagnostics GmbH, Mannheim, Germany). Blood gas values were corrected for temperature using the body temperature measured via the pulmonary artery catheter at the corresponding time point.

Cardiac output estimation was continuously generated every 20 seconds by the Vigilance II continuous cardiac output monitor, which uses an algorithm that integrates thermodilution data from the thermal filament of the pulmonary artery catheter with pressure-based CO derived from the pulmonary artery waveform.

### Data analysis

Data collected during the experiment were used to calculate outcome variables representing the oxygen cascade. Respiratory rate, TIV, minute tidal impedance variation (TIV_MIN_), and arterial partial pressure of carbon dioxide (PaCO_2_) were used to assess ventilation (Table 1). At each data collection time point, RR_EIT_ was calculated as the number of end-inspirations per minute from the EIT recordings. The TIV was calculated as the average total impedance change (∆Z) between the start and end of each inspiration [33]. The TIV was further multiplied by RR_EIT_ to provide a surrogate estimate of TIV_MIN_. Arterial partial pressure of carbon dioxide (PaCO_2_),directly measured by the automated blood gas analyser, was used for analysis.

To evaluate gas exchange efficiency, venous admixture (Qs/Qt) was calculated. The calculation was based on standard equations incorporating oxygen contents (Table 1). Arterial and mixed venous oxygen saturation (SaO_2_ and SvO_2_), used to determine oxygen content, were calculated from pH, partial pressure of carbon dioxide, and partial pressure of oxygen values based on an established algorithm for computing the oxygen dissociation curve in sheep [34]. Arterial partial pressure of oxygen (PaO_2_), determined using the blood gas analyser, was included in the analysis. Oxygen delivery (DO_2_) was calculated as the product of CO and CaO_2_, using the average CO recorded over one minute after the corresponding blood sampling and the calculated CaO_2_. Oxygen extraction ratio (ER) in percent was determined by dividing VO_2_ by DO_2_. Oxygen consumption (VO_2_) was calculated using the Fick principle, as the product of cardiac output and the arteriovenous difference in oxygen content (CaO_2_ – CvO_2_).

### Statistical analysis

Statistical analyses were performed using R software (version 4.2.2; R Core Team, 2022). Linear mixed-effects models were applied to the primary outcome variables including RR_EIT_, TIV, TIV_MIN_, PaCO_2_, Qs/Qt, PaO_2_, DO_2,_ and ER, using the lmer function from the lme4 package (version 1.1–35.3) [35]. Additional variables derived from data analysis were reported descriptively in supplementary materials.

The factor ‘treatment’ included three groups: BIMU-8, vatinoxan, and control, whereas ‘time’ was divided into four phases: BL, PE, PT, and PN, with the PT phase containing three distinct time points. The initial models included ‘treatment’, ‘time’ and their interaction as fixed effects. Sheep ID was included as a random effect to account for repeated measures. A significant interaction between treatment and time was observed for TIV and DO_2_. For the other variables, the interaction term (treatment*time) was not significant, and its removal improved the models as indicated by lower AICc values. Therefore, the interaction term was retained only for TIV and DO_2_, while for the remaining main variables, models included only ‘treatment’ and ‘time’ as fixed effects.

Model diagnostics included checks for dispersion and evaluation of simulated residuals using the *DHARMa* package (v.0.4.6) [36] to assess normality, heteroscedasticity, and overall distribution. Autocorrelation analysis was also performed to assess the independence of residuals.

Residuals for TIV and TIV_MIN_ were not normally distributed and were therefore transformed using Box-Cox transformations. Model fit was re-evaluated after transformation to confirm that model assumptions were met.

The significance of fixed effects was determined using F-tests from the regression model. These tests evaluated the effect of each predictor while accounting for the estimated denominator degrees of freedom (Satterthwaite approximation). Significance threshold was set at *p* = 0.05. We tested differences within the treatment and time point groups with post hoc pairwise comparisons using estimated marginal means (*emmeans*, v. 1.8.9) [37], with Tukey’s adjustment applied within each model to control for multiple testing.

## Results

### General results

All sheep (*n* = 6) achieved adequate immobilisation, as indicated by the absence of voluntary movement and responsiveness. Following etorphine administration, initial signs such as nystagmus, excitement, and muscle stiffness were observed within 3.7 ± 1.9 min. In the final trial, the experiment had to be discontinued in two sheep because cut-off criteria (as previously described in Rattanathanya et al., 2026) were reached, including a mean pulmonary arterial pressure exceeding 50 mmHg and apnoea lasting over 30 seconds, prior to completing the data collection timeline. Both animals recovered fully after naltrexone administration, with no subsequent complications. Therefore, only data collected before termination were included in the analysis, BL to T11 of PT from one sheep in the control treatment, and BL to PE from another sheep in the BIMU-8 treatment. One arterial blood gas sample collected after PN from one control-treated individual was excluded due to technical error and blood clotting. Means with standard deviations for each variable, together with the effects of time and treatment for each variable are presented in Table 2, and the full model outputs for each variable are provided in the supplementary material.

**Table 2:** Effects of treatment on outcome variables. Each variable presented in mean ± SD in four phases: baseline, post-etorphine, post-treatment, and post-naltrexone, in etorphine-immobilised sheep (*n* = 6). F-values for the effects of time and treatment on each variable, with corresponding *p*-values are presented. Statistical significance was set at *p* = 0.05

Variables		Treatment	Baseline	Post-etorphine	Post-treatment			Post-naltrexone	Effect of Time	Effect of Treatment
					T5	T11	T17			
*Ventilation*	RR_EIT_ (bpm)	BIMU-8	29 ± 5	15 ± 7	16 ± 7	17 ± 7	17 ± 7	31 ± 3	F_3,91_ = 66.67, *p* < 0.0001	F_2,92_ = 5.33, *p* = 0.006
		Vatinoxan	27 ± 6	12 ± 3	13 ± 2	19 ± 4	15 ± 4	28 ± 8		
		Control	29 ± 8	15 ± 5	15 ± 4	17 ± 6	19 ± 7	40 ± 8		
	TIV (AU)	BIMU-8	564 ± 191	403 ± 135	462 ± 175	481 ± 181	478 ± 166	740 ± 386	F_3,84_ = 44.31, *p* < 0.0001	F_2,84_ = 0.72, *p* = 0.49
		Vatinoxan	588 ± 205	462 ± 165	399 ± 184	387 ± 166	349 ± 169	690 ± 280		
		Control	610 ± 205	452 ± 166	417 ± 126	419 ± 123	460 ± 127	669 ± 188		
	TIV_MIN_ (AU/min)	BIMU-8	15,516 ± 3,821	5,771 ± 2,481	7,189 ± 3,240	7,647 ± 3,235	7,822 ± 3,604	21,936 ± 9,499	F_3,90_= 135.74, *p* < 0.0001	F_2,90_= 10.99, *p* < 0.0001
		Vatinoxan	15,495 ± 4,112	5,147 ± 1,349	5,184 ± 2,061	5,234 ± 2,220	4,882 ± 2,024	18,550 ± 5,504		
		Control	16,023 ± 4,761	7,277 ± 2,278	5,723 ± 996	6,697 ± 1,834	8,387 ± 3,633	26,160 ± 6,690		
	PaCO_2_ (mmHg)	BIMU-8	43.1 ± 4.2	51.0 ± 6.1	58.9 ± 4.3	61.5 ± 6.0	62.5 ± 4.9	45.1 ± 6.3	F_3,90_ = 124.48, *p* < 0.0001	F_2,90_ = 2.83, *p* = 0.064
		Vatinoxan	41.7 ± 2.8	54.9 ± 5.3	61.4 ± 4.5	64.4 ± 5.8	69.1 ± 3.8	46.3 ± 8.3		
		Control	41.3 ± 4.9	50.8 ± 8.2	59.7 ± 4.7	63.5 ± 5.5	63.9 ± 4.2	46.6 ± 5.3		
*Gas exchange*	Venous admixture	BIMU-8	0.13 ± 0.05	0.57 ± 0.29	0.7 ± 0.25	0.7 ± 0.23	0.62 ± 0.20	0.125 ± 0.05	F_3,90_ = 94.53, *p* < 0.0001	F_2,91_ = 5.48, *p* = 0.006
		Vatinoxan	0.1 ± 0.00	0.8 ± 0.06	0.88 ± 0.08	0.77 ± 0.15	0.85 ± 0.08	0.23 ± 0.08		
		Control	0.12 ± 0.04	0.5 ± 0.32	0.77 ± 0.02	0.77 ± 0.02	0.7 ± 0.19	0.34 ± 0.21		
*Arterial partial pressure of oxygen*	PaO_2_ (mmHg)	BIMU-8	94.0 ± 5.5	52.2 ± 21.6	40.8 ± 18.6	42.2 ± 18.1	46.0 ± 16.7	95.6 ± 10.3	F_3,90_ = 104.82, *p* < 0.0001	F_2,91_ = 4.33, *p* = 0.016
		Vatinoxan	96.9 ± 6.4	38.5 ± 5.8	30.3 ± 7.8	35.6 ± 9.1	32.4 ± 9.1	77.2 ± 14.0		
		Control	96.6 ± 7.9	57.0 ± 26.0	37.2 ± 13.2	38.3 ± 13.3	44.2 ± 11.4	73.5 ± 26.9		
*Oxygen delivery*	CaO_2_ (mL/dL)	BIMU-8	13.0 ± 1.1	8.5 ± 4.1	6.4 ± 4.5	6.3 ± 4.7	6.9 ± 4.3	16.1 ± 0.7	F_3,90_ = 64.66, *p* < 0.0001	F_2,90_ = 2.30, *p* = 0.106
		Vatinoxan	13.1 ± 1.1	6.3 ± 2.0	3.8 ± 1.7	5.4 ± 3.3	4.0 ± 1.7	16.0 ± 1.6		
		Control	12.7 ± 1.5	9.3 ± 5.2	5.5 ± 3.7	5.5 ± 3.1	6.3 ± 2.4	13.2 ± 2.1		
	DO_2_ (mL/min)	BIMU-8	951 ± 307	650 ± 334	471 ± 376	485 ± 283	526 ± 423	1,219 ± 372	F_3,84_ = 51.55, *p* < 0.0001	F_2,85_ = 2.17, *p* = 0.12
		Vatinoxan	1,008 ± 165	473 ± 126	328 ± 159	494 ± 249	415 ± 193	1,792 ± 448		
		Control	921 ± 241	626 ± 332	387 ± 224	416 ± 189	493 ± 152	1,119 ± 227		
*Oxygen extraction ratio*	ER	BIMU-8	0.42 ± 0.15	0.48 ± 0.08	0.62 ± 0.08	0.64 ± 0.09	0.60 ± 0.12	0.45 ± 0.06	F_3,91_ = 8.09, *p* < 0.0001	F_2,92_ = 1.43, *p* = 0.25
		Vatinoxan	0.43 ± 0.05	0.4 ± 0.11	0.48 ± 0.08	0.62 ± 0.17	0.55 ± 0.10	0.48 ± 0.13		
		Control	0.43 ± 0.10	0.50 ± 0.09	0.50 ± 0.13	0.52 ± 0.15	0.52 ± 0.11	0.50 ± 0.1		

### Effects on ventilation

**Fig. 3:**
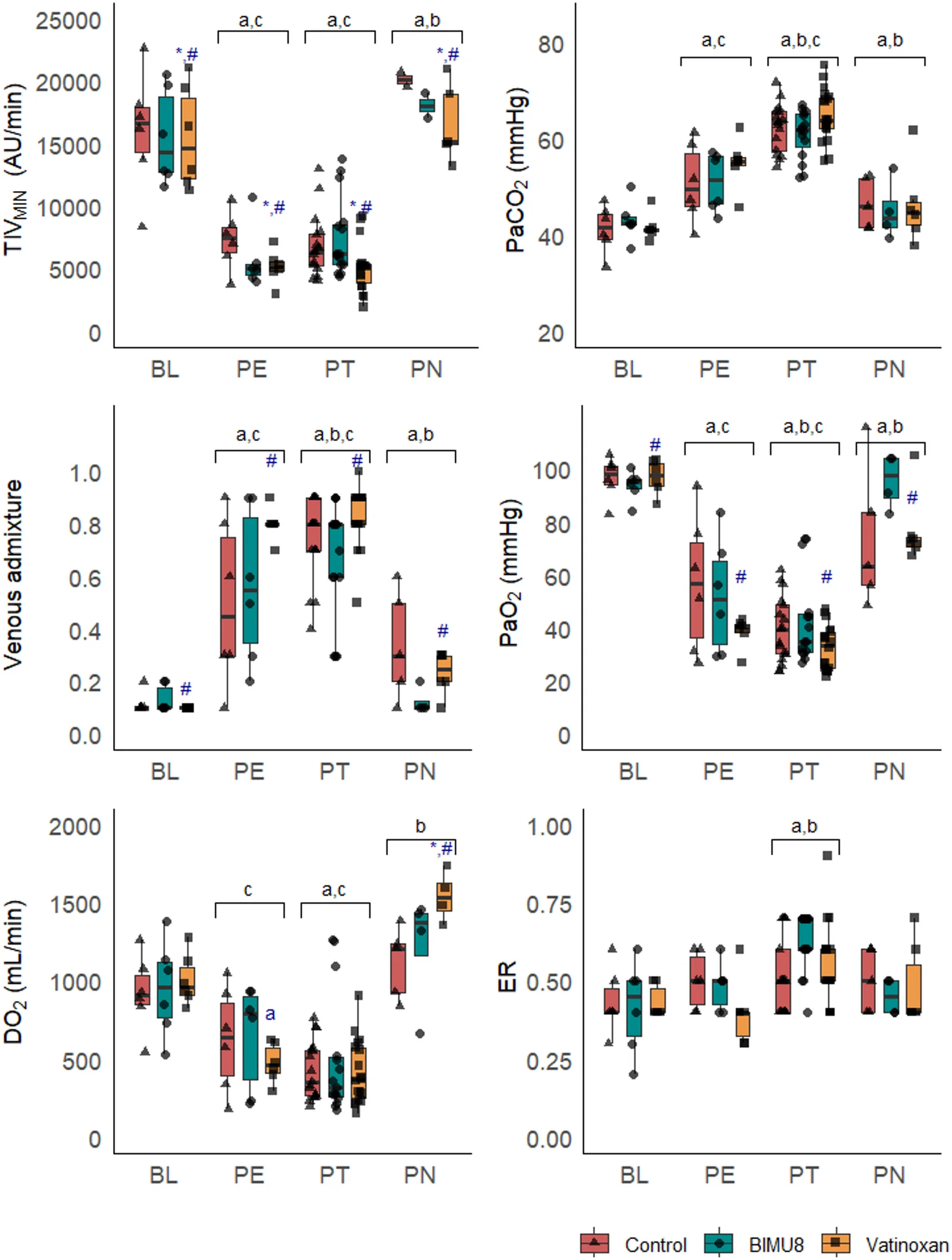
Effects of time and treatment on outcome variables. All variables, minute tidal impedance variation (TIV_MIN_), arterial partial pressure of carbon dioxide (PaCO_2_), venous admixture (Qs/Qt), arterial partial pressure of oxygen (PaO_2_), oxygen delivery (DO_2_) and oxygen extraction ratio (ER), are presented in four phases: baseline (BL), post-etorphine (PE), post-treatment (PT), and post-naltrexone (PN), in etorphine-immobilised sheep (*n* = 6) receiving either sterile water (control), BIMU-8, or vatinoxan. Values are presented as box plots with individual data points overlaid (▲ for control, ● for BIMU-8, ■ for vatinoxan). The box plots represent the median (horizontal line), interquartile range (IQR), and whiskers extending to 1.5 x IQR (a) *p* < 0.05, significantly different from baseline. (b) *p* < 0.05, significantly different from post-etorphine. (c) *p* < 0.05, significantly different from post-naltrexone (*) *p* < 0.05, significantly different from control across all time points (#) *p* < 0.05, significantly different BIMU-8 vs. vatinoxan across all time points

Significant effects of time on RR_EIT_, TIV, TIV_MIN_, and PaCO_2_ were observed (Table 2). Over time, RR_EIT_, TIV, and TIV_MIN_ decreased, whereas PaCO_2_ increased. After etorphine administration, RR_EIT_ significantly decreased from BL to PE (*p* < 0.01) and remained below BL levels throughout the immobilisation period (*p* < 0.01) in all treatments. Following naltrexone administration, RR_EIT_ returned to BL levels. TIV was also decreased after etorphine, but patterns varied across treatments. In BIMU-8 treatment, TIV decreased from BL after etorphine injection (PE, *p* < 0.01), but was no longer significantly different from BL after BIMU-8 administration (PT, *p* = 0.29) and increased beyond both BL and PE after PN (*p* < 0.01 for both comparisons). In contrast, vatinoxan led to a decrease in TIV during PT relative to BL (*p* < 0.01) and PE (*p* = 0.02), and returned to BL after PN. Consequently, in all treatments, TIV_MIN_ decreased by 60% from BL (*p* < 0.01) and remained lower than BL during PT (*p* < 0.01), while PaCO_2_ increased progressively over time (Fig. 3). After naltrexone administration, TIV_MIN_ increased and exceeded BL values (*p* < 0.01), while PaCO_2_ decreased and was lower than during the PE and PT phases (*p* < 0.01) but remained higher than BL (*p* = 0.04).

Treatment with vatinoxan significantly affected RR_EIT_ and TIV_MIN_, but not PaCO_2_ (Table 2). Vatinoxan induced lower RR_EIT_ compared to the control treatment (*p* < 0.01) and lower TIV_MIN_ compared to both BIMU-8 and control treatment (*p* < 0.01). In addition, a significant interaction between treatment and time was observed for TIV (F_6,84_ = 3.40, *p* = 0.005). TIV was lower in the vatinoxan compared to both the BIMU-8 and control treatment (*p* < 0.001 and *p* = 0.02, respectively) during the PT period. However, treatment had no overall effect on PaCO_2_, and the sheep remained hypercapnic (PaCO_2_ > 45 mmHg) in all treatments during immobilisations.

### Effects on gas exchange

Venous admixture (Qs/Qt) increased over time (Table 2), and exceeded BL after etorphine administration (PE, *p* < 0.01). During PT, Qs/Qt was higher than at both BL (*p* < 0.01) and PE (*p* = 0.02) (Fig. 3). After naltrexone was injected, Qs/Qt returned to BL levels at PN. Treatment had a significant effect on Qs/Qt, with BIMU-8 inducing lower Qs/Qt compared to vatinoxan (*p* < 0.01). However, neither BIMU-8 nor vatinoxan showed a statistically significant difference in Qs/Qt compared to the control treatment.

### Effects on arterial oxygen partial pressure (PaO_2_)

Etorphine induced severe hypoxaemia (PaO_2_ < 60 mmHg) in all treatments (Fig. 3). Following etorphine injection, PaO_2_ declined significantly at PE compared to BL (*p* < 0.01). During PT, PaO_2_ fell further and was lower than both BL (*p* < 0.01) and PE (*p* = 0.02). PaO_2_ remained below baseline at PN (*p* = 0.01). Significant differences were found between BIMU-8 and vatinoxan treatments (Table 2), with BIMU-8 inducing higher values in PaO_2_ (*p* = 0.01). However, neither BIMU-8 nor vatinoxan showed significant different from control, and hypoxaemia persisted in all treatments.

### Effects on oxygen content and delivery

Arterial oxygen content decreased over time across all treatments, reaching its lowest level during PT (Table 2) (*p* < 0.01 compared with BL and PE). The decreased CaO_2_ returned to BL levels after naltrexone administration. Neither BIMU-8 nor vatinoxan had a significant effect on the reduction in CaO_2_ induced by etorphine.

A significant interaction between treatment and time was observed (F_3,84_= 3.4, *p* = 0.005), with DO_2_ in the vatinoxan treatment being significantly higher than in control and BIMU-8 after naltrexone injection (PN) (Fig. 3). In both the control and BIMU-8 treatments, DO_2_ decreased over time, with significant values below BL observed during PT (*p* < 0.01). However, following naltrexone administration, DO_2_ returned to BL levels and was higher than during the PE and PT periods in both treatments (*p* < 0.05 for all comparisons). In contrast, DO_2_ in vatinoxan treatment was lower than BL at both PE and PT (*p* < 0.01). During the PN period, DO_2_ in the vatinoxan treatment was higher than in all other phases, including BL, and higher than in both the control and BIMU-8 treatments (*p* < 0.01 for all comparisons).

### Oxygen extraction ratio (ER)

Oxygen extraction ratio was significantly affected by time, but not by treatment (Table 2). Compared to BL and PE, the extraction ratio was significantly higher during PT period (*p* < 0.01), but returned to BL after naltrexone injection (Fig. 3).

## Discussion

Etorphine immobilisation impaired multiple sequential steps of the oxygen cascade, not only a single isolated component. It caused disturbances in ventilation, gas exchange followed by reduced oxygen delivery and compensatory increase in oxygen extraction in sheep. Neither BIMU-8 nor vatinoxan had significant effects on these variables, and the animals remained hypoxaemic and hypoxic in all treatments. Naltrexone normalised most variables, indicating that the observed changes were mediated by opioid receptor activation.

### Etorphine with control treatment

Respiratory depression and hypoventilation were indicated by bradypnea and a reduction in TIV resulting in a tremendous decrease in TIV_MIN_, and hypercapnia compared to BL. These findings were consistent with reductions in respiratory rates and tidal volumes, and minute ventilation reported in goats and African buffalo immobilised using etorphine combinations [38, 39].

Alongside effects on ventilation, venous admixture increased markedly over time after etorphine administration, reflecting impaired gas exchange, causing a greater proportion of deoxygenated blood entering the arterial circulation. Limited gas exchange induced by etorphine has been reported to be associated with ventilation-perfusion mismatch and pulmonary hypertension, possibly driven by sympathetic upregulation [12, 18]. In etorphine-immobilised animals, pulmonary hypertension may elevate pulmonary capillary hydrostatic pressure, resulting in accumulation of extravascular lung fluid, increased diffusion distance, and impaired oxygen diffusion across the alveolar-blood barrier contributing to hypoxaemia [11, 13]. In addition, hypoxaemia may also induce hypoxic pulmonary vasoconstriction, a physiological response that redistributes pulmonary blood flow but may further increase pulmonary vascular resistance and exacerbate pulmonary hypertension.

The impairment in gas exchange caused a decrease in oxygen content. Therefore, although CO remained unchanged [18], the observed reduction in DO_2_ was primarily attributable to decreased CaO_2_. Oxygen extraction, representing the proportion of oxygen consumption relative to delivery, increased over time during immobilisation indicating compensatory tissue adaptation to the reduced oxygen delivery within the oxygen cascade. This finding aligns with reports in rhinoceroses, where animals exhibited increased ER likely due to a combination of reduced oxygen delivery and heightened oxygen demand in active muscle tissues as etorphine induces muscle tremors in this species [40]. In our study, muscle stiffness was observed but tremors did not appear to occur in any of the sheep.

### Etorphine with BIMU-8

After BIMU-8 administration, respiratory depression and hypoventilation persisted, in a similar way to the control treatment. These findings contrast with those in etorphine-immobilised goats, where BIMU-8 increased respiratory drive [17]. However, compared to the goat study, the dose of BIMU-8 used in the sheep was halved due to dysrhythmias observed in sheep, consistent with the compound’s known proarrhythmic effects mediated via cardiac 5-HT4 receptor activation or ion channel blockade [41, 42]. Therefore, BIMU-8’s effect on respiratory drive cannot be excluded, as higher doses were not tested [18].

Although BIMU-8 did not improve ventilation as expected, its ability to alleviate pulmonary hypertension has been reported in sheep [18]. However, venous admixture remained high and comparable to the control treatment, indicating that BIMU-8 did not overcome the persistent hypoventilation and impaired gas exchange. These findings contrast with results from a study in etorphine-immobilised goats where improvement of both minute ventilation and narrowing of the gradient alveolar-arterial partial pressure of oxygen were observed after receiving BIMU-8 [17].

Although venous admixture did not decrease after BIMU-8 compared to control, it was lower than that observed with vatinoxan. However, BIMU-8 had no significant effect on oxygen content and therefore DO_2_, as cardiac output remained also unchanged [18]. These findings indicate that the effects of BIMU-8 were insufficient to achieve meaningful improvements in the oxygen cascade to effectively mitigate etorphine-induced respiratory compromise in sheep.

### Etorphine with vatinoxan

Vatinoxan decreased respiratory rates, TIV, and TIV_MIN_ even further compared to control. These effects were unexpected, and there is a lack of similar previous findings or a known underlying mechanism. Previous studies have reported no significant impact of vatinoxan on ventilation [25–27]. Furthermore, there were no significant differences in PaCO_2_ between the treatments in the sheep.

Vatinoxan may cause vasodilation and reduce vascular resistance in the pulmonary vasculature in etorphine immobilised sheep [18], which may contribute to improved gas exchange. However, the degree of venous admixture remained high, and was significantly greater than in the BIMU-8 treatment. This may be explained by the extent of hypoventilation in combination with incompletely resolved pulmonary hypertension following vatinoxan treatment, resulting in degrees of ventilation-perfusion mismatch that remained similar to the control but more severe than that observed with BIMU-8.

Because oxygen content did not rise after vatinoxan administration, and the improvement in CO induced by vatinoxan during immobilisation was not equivalent, no increase in DO_2_ was observed. Thus, although vatinoxan may promote pulmonary vasodilation and enhance CO, hypoventilation and ventilation-perfusion mismatch predominated causing severe hypoxaemia, limiting oxygen delivery to tissues in a similar way to the control.

### Future perspective

The potential of new agents to prevent hypoxaemia and hypoxia when combined with etorphine, as well as other complementary approaches, should be further investigated. While BIMU-8 and vatinoxan modulated specific components of the respiratory and cardiovascular responses, neither agent substantially improved hypoxaemia or hypoxia. This suggests that these adverse effects of etorphine might not be mitigated solely via one mechanism. Future research should focus on identifying the sequence and relative importance of the contributing mechanisms leading to hypoxaemia and hypoxia during etorphine immobilisation, as understanding the relative contributions of hypoventilation, pulmonary hypertension, ventilation-perfusion mismatch and other mechanisms could better guide targeted interventions. By clarifying which processes occur first and which are most critical, it may be possible to develop pharmacological or supportive strategies that more effectively counteract the adverse respiratory and cardiovascular effects of etorphine, ultimately enhancing both animal welfare and safety during wildlife immobilisation.

## Conclusion

Etorphine impairs all sequential steps of the oxygen cascade, and neither BIMU-8 nor vatinoxan mitigated any of the changes to a clinically relevant degree. Future research should elucidate the sequence and relative contributions of mechanisms underlying etorphine-induced respiratory compromise to support the development of more targeted interventions.

## Supplementary Information

Supplementary Material 1.

## Data Availability

The datasets used and/or analysed during the current study are available from the corresponding author on reasonable request.

## Abbreviations

PBC: Pre-Bötzinger complex
PaO_2_: Arterial partial pressure of oxygen
CO: Cardiac output
DO_2_: Oxygen delivery
VO_2_: Oxygen consumption
ER: Oxygen extraction ratio
5-HT4: 5-hydroxytryptamine 4
CaO_2_: Arterial oxygen content
EIT: Electrical impedance tomography
RR_EIT_: Respiratory rate
TIV: Tidal impedance variation
BL: Baseline
PE: Post-etorphine
PT: Post-treatment
PN: Post-naltrexone
AU: Arbitrary units
TIV_MIN_: Minute tidal impedance variation
PaCO_2_: Arterial partial pressure of carbon dioxide
△Z: Total impedance change
Qs/Qt: Venous admixture
SaO_2_: Arterial oxygen saturation
SvO_2_: Mixed venous oxygen saturation
CvO_2_: Mixed venous oxygen content
