# Supplementary material for: Effects of a selective 5-HT4 agonist and an alpha-2 adrenoceptor antagonist on etorphine-induced impairment across the oxygen cascade in immobilised sheep (Ovis aries): a randomised, prospective, and controlled trial
Source: BMC Vet Res. 2026 Aug 7;22:666. doi: 10.1186/s12917-026-05790-4 (PMC13733009; doi:10.1186/s12917-026-05790-4)
Supplement: Supplementary file 1 [file 12917_2026_5790_MOESM1_ESM.docx]

**Supplementary Materials**

**Supplementary material 1: Additional variables**

**Table 1: additional variables from data analysis are presented in mean ± SD.**

| **Variables** | **treatment** | **Baseline** | **Post-etorphine** | **Post-treatment** | | | **Post-naltrexone** |
| --- | --- | --- | --- | --- | --- | --- | --- |
|  |  |  |  | **T6** | **T12** | **T18** |  |
| **SaO_2_ (%)** | **BIMU-8** | 93.85 ± 2.53 | 57.89 ± 30.14 | 38.92 ± 27.27 | 39.39 ± 25.55 | 37.89 ± 28.01 | 74.44 ± 41.72 |
|  | **Vatinoxan** | 94.92 ± 1.35 | 40.70 ± 12.03 | 23.66 ± 12.04 | 21.92 ± 15.98 | 25.07 ± 14.49 | 86.42 ± 5.45 |
|  | **Control** | 94.46 ± 2.19 | 61.29 ± 32.57 | 34.93 ± 22.54 | 35.83 ± 22.55 | 44.40 ± 19.33 | 79.10 ± 16.26 |
| **PvO_2_ (mmHg)** | **BIMU-8** | 44.70 ± 6.81 | 31.88 ± 7.58 | 23.74 ± 11.08 | 24.42 ± 10.09 | 27.52 ± 10.94 | 44.28 ± 3.92 |
|  | **Vatinoxan** | 43.60 ± 2.38 | 29.92 ± 6.20 | 22.88 ± 6.93 | 20.73 ± 8.60 | 21.8 ± 8.05 | 44.16 ± 4.85 |
|  | **Control** | 44.33 ± 4.48 | 32.22 ± 11.01 | 25.62 ± 9.62 | 25.30 ± 10.82 | 29.74 ± 7.41 | 40.06 ± 5.85 |
| **PvCO_2_ (mmHg)** | **BIMU-8** | 48.63 ± 4.12 | 53.13 ± 4.28 | 61.60 ± 3.27 | 65.32 ± 2.62 | 66.94 ± 4.24 | 57.88 ± 6.03 |
|  | **Vatinoxan** | 47.53 ± 3.26 | 55.75 ± 3.58 | 61.72 ± 2.21 | 66.95 ± 4.24 | 71.03 ± 3.58 | 59.98 ± 11.85 |
|  | **Control** | 47.37 ± 5.06 | 53.33 ± 7.68 | 61.05 ± 5.09 | 65.98 ± 5.11 | 66.38 ± 3.29 | 59.76 ± 5.12 |
| **SvO_2_ (%)** | **BIMU-8** | 54.42 ± 13.46 | 29.72 ± 14.42 | 16.06 ± 14.27 | 15.51 ± 13.68 | 19.23 ± 15.56 | 47.85 ± 60.1 |
|  | **Vatinoxan** | 54.16 ± 5.78 | 25.09 ± 8.90 | 13.47 ± 7.28 | 11.60 ± 8.22 | 12.07 ± 8.52 | 39.41 ± 22.09 |
|  | **Control** | 54.13 ± 9.65 | 31.85 ± 19.88 | 18.025 ± 14.01 | 17.59 ± 13.95 | 21.65 ± 11.17 | 39.56 ± 12.29 |
| **CvO_2_ (mL/dL)** | **BIMU-8** | 7.65 ± 2.08 | 4.45 ± 2.12 | 2.7 ± 2.56 | 2.56 ± 2.55 | 3.02 ± 2.84 | 8.2 ± 1.16 |
|  | **Vatinoxan** | 7.47 ± 1.28 | 3.92 ± 1.43 | 2.02 ± 1.07 | 1.83 ± 1.12 | 1.92 ± 1.12 | 8.17 ± 1.96 |
|  | **Control** | 7.28 ± 1.59 | 4.75 ± 2.88 | 2.85 ± 2.22 | 2.75 ± 2.03 | 3.14 ± 1.56 | 6.64 ± 1.85 |
| **C_(a-v)_O_2_ (mL/dL)** | **BIMU-8** | 5.32 ± 1.57 | 4.08 ± 2.24 | 3.7 ± 2.22 | 3.66 ± 2.21 | 3.8 ± 1.57 | 7.45 ± 0.64 |
|  | **Vatinoxan** | 5.67 ± 0.41 | 2.43 ± 0.68 | 1.8 ± 0.67 | 3.6 ± 3.41 | 2.07 ± 0.79 | 7.77 ± 2.22 |
|  | **Control** | 5.45 ± 1.07 | 4.57 ± 2.72 | 2.67 ± 1.54 | 2.75 ± 1.18 | 3.14 ± 0.98 | 6.56 ± 1.40 |
| **CcO_2_ (mL/dL)** | **BIMU-8** | 13.83 ± 0.93 | 15.2 ±1.28 | 16.54 ± 2.03 | 15.44 ± 1.83 | 14.7 ±1.68 | 17.35 ± 1.08 |
|  | **Vatinoxan** | 13.87 ± 1.11 | 15.55 ± 1.20 | 16.8 ± 2.67 | 17 ± 3.48 | 16.97 ± 3.47 | 18.55 ± 2.01 |
|  | **Control** | 13.48 ± 1.42 | 14.9 ± 1.73 | 15.57 ± 1.60 | 16.2 ± 3.18 | 14.64 ± 2.62 | 16.92 ± 2.08 |
| **VO_2_ (mL/min)** | **BIMU-8** | 397.6 ± 195.9 | 307.9 ± 167.6 | 270.4 ± 179.0 | 283.3 ± 210.4 | 284.7 ± 156.8 | 561.1 ± 174.4 |
|  | **Vatinoxan** | 439.9 ± 103.1 | 183.1 ± 42.7 | 154.8 ± 64.6 | 317.2 ± 249.9 | 210.6 ± 78.3 | 894.8 ± 428.9 |
|  | **Control** | 388.3 ± 95.6 | 301.4 ± 144.9 | 187.3 ± 93.9 | 209.6 ± 71.2 | 248.4 ± 65.1 | 561.6 ± 160.6 |

**Supplementary material 2: Model results**

**Respiratory rate (RR_EIT_)**

Model: lmer (RR_EIT_ ~ treatment + time + (1|Sheep.ID), data=data)

Random effects:

| Groups | Name | Variance | Std.Dev. |
| --- | --- | --- | --- |
| Sheep.ID | (Intercept) | 6.805 | 2.609 |
| Residual |  | 27.432 | 5.238 |

Fixed effects:

|  | Estimate | Std.Error | df | t value | Pr(>\|t\|) |  |
| --- | --- | --- | --- | --- | --- | --- |
| (Intercept) | 30.109 | 1.789 | 24.012 | 16.827 | <.0001 | *** |
| treatmentBIMU8 | -1.6 | 1.301 | 91.864 | -1.23 | 0.22196 |  |
| treatmentVatinoxan | -4.059 | 1.255 | 91.19 | -3.236 | 0.00169 | ** |
| timepost etorphine | -14.333 | 1.746 | 91.075 | -8.21 | <.0001 | *** |
| timepost naltrexone | 4.653 | 1.802 | 91.183 | 2.582 | 0.01142 | * |
| timepost treatment | -12.594 | 1.442 | 91.19 | -8.734 | <.0001 | *** |

Signif. codes: 0 '***' 0.001 '**' 0.01 '*' 0.05 '.' 0.1 ' ' 1

Type III Analysis of Variance Table with Satterthwaite's method:

|  | Sum Sq | Mean Sq | NumDF | DenDF | F value | Pr(>F) |  |
| --- | --- | --- | --- | --- | --- | --- | --- |
| treatment | 292.5 | 146.26 | 2 | 91.572 | 5.3317 | 0.006451 | ** |
| time | 5486.3 | 1828.75 | 3 | 91.159 | 66.6660 | <.0001 | *** |

Signif. codes: 0 '***' 0.001 '**' 0.01 '*' 0.05 '.' 0.1 ' ' 1

Pairwise comparison of time

| contrast | estimate | SE | df | t.ratio | p.value |
| --- | --- | --- | --- | --- | --- |
| baseline - post etorphine | 14.33 | 1.75 | 91.0 | 8.210 | <.0001 |
| baseline - post naltrexone | -4.65 | 1.80 | 91.1 | -2.581 | 0.0547 |
| baseline - post treatment | 12.59 | 1.44 | 91.1 | 8.732 | <.0001 |
| post etorphine - post naltrexone | -18.99 | 1.80 | 91.1 | -10.532 | <.0001 |
| post etorphine - post treatment | -1.74 | 1.44 | 91.1 | -1.206 | 0.6248 |
| post naltrexone - post treatment | 17.25 | 1.51 | 91.0 | 11.456 | <.0001 |

Results are averaged over the levels of treatment

Degrees-of-freedom method: kenward-roger

P value adjustment: Tukey method for comparing a family of 4 estimates

Pairwise comparison of treatment

| contrast | estimate | SE | df | t.ratio | p.value |
| --- | --- | --- | --- | --- | --- |
| Control - BIMU8 | 1.60 | 1.30 | 91.8 | 1.227 | 0.4402 |
| Control - Vatinoxan | 4.06 | 1.25 | 91.1 | 3.235 | 0.0048 |
| BIMU8 - Vatinoxan | 2.46 | 1.28 | 91.6 | 1.917 | 0.1398 |

Results are averaged over the levels of time

Degrees-of-freedom method: kenward-roger

P value adjustment: tukey method for comparing a family of 3 estimates

**Tidal impedance variation (TIV)**

**Model: lmer (TIV~ treatment*time + (1|Sheep.ID), data=data)**

Random effects:

| Groups |  | Name | Variance | Std.Dev. |
| --- | --- | --- | --- | --- |
| Sheep.ID |  | (Intercept) | 0.573 | 0.757 |
| Residual |  |  | 0.1918 | 0.4379 |

Fixed effects:

|  | Estimate | Std. Error | df | t value | Pr(>\|t\|) | signif |
| --- | --- | --- | --- | --- | --- | --- |
| (Intercept) | 0.59783 | 0.35702 | 8.52077 | 1.675 | 0.13025 |  |
| treatmentBIMU8 | -0.20022 | 0.25284 | 84.00783 | -0.792 | 0.43066 |  |
| treatmentVatinoxan | -0.07168 | 0.25284 | 84.00783 | -0.283 | 0.77751 |  |
| timepost etorphine | -0.81583 | 0.25284 | 84.00783 | -3.227 | 0.00179 | ** |
| timepost naltrexone | 0.20405 | 0.26593 | 84.02764 | 0.767 | 0.44505 |  |
| timepost treatment | -0.91742 | 0.20804 | 84.01063 | -4.41 | <.0001 | *** |
| treatmentBIMU8:timepost etorphine | -0.07516 | 0.35757 | 84.00783 | -0.21 | 0.83402 |  |
| treatmentVatinoxan:timepost etorphine | 0.17424 | 0.35757 | 84.00783 | 0.487 | 0.62733 |  |
| treatmentBIMU8:timepost naltrexone | 0.61165 | 0.38998 | 84.05305 | 1.568 | 0.12055 |  |
| treatmentVatinoxan:timepost naltrexone | 0.15717 | 0.36694 | 84.01824 | 0.428 | 0.66952 |  |
| treatmentBIMU8:timepost treatment | 0.54087 | 0.29755 | 84.03244 | 1.818 | 0.07267 | . |
| treatmentVatinoxan:timepost treatment | -0.34509 | 0.29309 | 84.00924 | -1.177 | 0.24234 |  |

Signif. codes: 0 '***' 0.001 '**' 0.01 '*' 0.05 '.' 0.1 ' ' 1

Type III Analysis of Variance Table with Satterthwaite's method:

|  | Sum sq | Mean Sq | NumDF | DenDF | F value | Pr(>F) |  |
| --- | --- | --- | --- | --- | --- | --- | --- |
| treatment | 0.2749 | 0.1374 | 2 | 84.044 | 0.7166 | 0.491375 |  |
| time | 25.4923 | 8.4974 | 3 | 84.02 | 44.3064 | <.0001 | *** |
| treatment:time | 3.9153 | 0.6526 | 6 | 84.024 | 3.4025 | 0.004703 | ** |

Pairwise comparison of time across treatment:

*Treatment = control*

| Contrast | Estimate | SE | df | t.ratio | p.value |
| --- | --- | --- | --- | --- | --- |
| baseline – post etorphine | 0.816 | 0.253 | 84 | 3.227 | 0.0095 |
| baseline – post naltrexone | -0.204 | 0.266 | 84 | -0.767 | 0.869 |
| baseline – post treatment | 0.917 | 0.208 | 84 | 4.41 | 0.0002 |
| post etorphine – post naltrexone | -1.02 | 0.266 | 84 | -3.835 | 0.0014 |
| post etorphine – post treatment | 0.102 | 0.208 | 84 | 0.488 | 0.9615 |
| post naltrexone – post treatment | 1.121 | 0.223 | 84 | 5.023 | <.0001 |

*Treatment = BIMU-8*

| Contrast | Estimate | SE | df | t.ratio | p.value |
| --- | --- | --- | --- | --- | --- |
| baseline – post etorphine | 0.891 | 0.253 | 84 | 3.524 | 0.0038 |
| baseline – post naltrexone | -0.816 | 0.285 | 84 | -2.867 | 0.0264 |
| baseline – post treatment | 0.377 | 0.213 | 84 | 1.771 | 0.2945 |
| post etorphine – post naltrexone | -1.707 | 0.285 | 84 | -5.999 | <.0001 |
| post etorphine – post treatment | -0.514 | 0.213 | 84 | -2.42 | 0.0811 |
| post naltrexone – post treatment | 1.192 | 0.248 | 84 | 4.815 | <.0001 |

*Treatment = Vatinoxan*

| Contrast | Estimate | SE | df | t.ratio | p.value |
| --- | --- | --- | --- | --- | --- |
| baseline – post etorphine | 0.642 | 0.253 | 84 | 2.538 | 0.0614 |
| baseline – post naltrexone | -0.361 | 0.253 | 84 | -1.429 | 0.4853 |
| baseline – post treatment | 1.263 | 0.206 | 84 | 6.115 | <.0001 |
| post etorphine – post naltrexone | -1.003 | 0.253 | 84 | -3.966 | 0.0009 |
| post etorphine – post treatment | 0.621 | 0.206 | 84 | 3.008 | 0.0179 |
| post naltrexone – post treatment | 1.624 | 0.206 | 84 | 7.865 | <.0001 |

Degrees-of-freedom method: kenward-roger

P value adjustment: tukey method for comparing a family of 4 estimates

Pairwise comparison of treatment across time:

*Baseline*

| Contrast | Estimate | SE | df | t.ratio | p.value |
| --- | --- | --- | --- | --- | --- |
| Control – BIMU-8 | 0.2002 | 0.253 | 84 | 0.792 | 0.709 |
| Control – Vatinoxan | 0.0717 | 0.253 | 84 | 0.283 | 0.9567 |
| BIMU-8 – Vatinoxan | -0.1285 | 0.253 | 84 | -0.508 | 0.8675 |

*Post etorphine*

| Contrast | Estimate | SE | df | t.ratio | p.value |
| --- | --- | --- | --- | --- | --- |
| Control – BIMU-8 | 0.2754 | 0.253 | 84 | 1.089 | 0.5234 |
| Control – Vatinoxan | -0.1026 | 0.253 | 84 | -0.406 | 0.9134 |
| BIMU-8 – Vatinoxan | -0.3779 | 0.253 | 84 | -1.495 | 0.2986 |

*Post treatment*

| Contrast | Estimate | SE | df | t.ratio | p.value |
| --- | --- | --- | --- | --- | --- |
| Control – BIMU-8 | -0.3407 | 0.157 | 84.1 | -2.171 | 0.0821 |
| Control – Vatinoxan | 0.4168 | 0.148 | 84 | 2.812 | 0.0167 |
| BIMU-8 – Vatinoxan | 0.7574 | 0.155 | 84.1 | 4.9 | <.0001 |

*Post naltrexone*

| Contrast | Estimate | SE | df | t.ratio | p.value |
| --- | --- | --- | --- | --- | --- |
| Control – BIMU-8 | -0.4114 | 0.297 | 84.1 | -1.385 | 0.3531 |
| Control – Vatinoxan | -0.0855 | 0.266 | 84 | -0.321 | 0.9447 |
| BIMU-8 – Vatinoxan | 0.3259 | 0.285 | 84 | 1.146 | 0.489 |

Degrees-of-freedom method: kenward-roger

P value adjustment: tukey method for comparing a family of 4 estimates

**Minute tidal impedance variation (TIV_min_)**

Model: lmer (TIV_min_~ treatment + time + (1|Sheep.ID))

Random effects:

| Groups | Name | Variance | Std.Dev. |
| --- | --- | --- | --- |
| Sheep.ID | (Intercept) | 0.1056 | 0.325 |
| Residual |  | 0.1723 | 0.4151 |

Fixed effects:

|  | Estimate | Std.Error | df | t value | Pr(>\|t\|) |  |
| --- | --- | --- | --- | --- | --- | --- |
| (Intercept) | 1.05558 | 0.17498 | 12.42697 | 6.033 | <.0001 | *** |
| treatmentBIMU8 | -0.02808 | 0.10415 | 90.44444 | -0.27 | 0.788 |  |
| treatmentVatinoxan | -0.41808 | 0.09944 | 90.11069 | -4.204 | <.0001 | *** |
| timepost etorphine | -1.5192 | 0.13836 | 90.05801 | -10.98 | <.0001 | *** |
| timepost naltrexone | 0.60409 | 0.14551 | 90.11816 | 4.151 | <.0001 | *** |
| timepost treatment | -1.44581 | 0.11429 | 90.11222 | -12.65 | <.0001 | *** |

Signif. codes: 0 '***' 0.001 '**' 0.01 '*' 0.05 '.' 0.1 ' ' 1

Type III Analysis of Variance Table with Satterthwaite's method:

|  | Sum Sq | Mean Sq | NumDF | DenDF | F value | Pr(>F) |  |
| --- | --- | --- | --- | --- | --- | --- | --- |
| treatment | 3.785 | 1.8925 | 2 | 90.296 | 10.985 | <.0001 | *** |
| time | 70.157 | 23.3857 | 3 | 90.105 | 135.741 | <.0001 | *** |

Signif. codes: 0 '***' 0.001 '**' 0.01 '*' 0.05 '.' 0.1 ' ' 1

Pairwise comparison of time

| contrast | estimate | SE | df | t.ratio | p.value |
| --- | --- | --- | --- | --- | --- |
| baseline - post etorphine | 1.5192 | 0.138 | 90 | 10.98 | <.0001 |
| baseline - post naltrexone | -0.6041 | 0.146 | 90.1 | -4.151 | 0.0004 |
| baseline - post treatment | 1.4458 | 0.114 | 90.1 | 12.649 | <.0001 |
| post etorphine - post naltrexone | -2.1233 | 0.146 | 90.1 | -14.589 | <.0001 |
| post etorphine - post treatment | -0.0734 | 0.114 | 90.1 | -0.642 | 0.9181 |
| post naltrexone - post treatment | 2.0499 | 0.122 | 90 | 16.744 | <.0001 |

Results are averaged over the levels of treatment

Degrees-of-freedom method: kenward-roger

P value adjustment: Tukey method for comparing a family of 4 estimates

Pairwise comparison of treatment

| contrast | estimate | SE | df | t.ratio | p.value |
| --- | --- | --- | --- | --- | --- |
| Control - BIMU8 | 0.0281 | 0.1042 | 90.4 | 0.269 | 0.9608 |
| Control - Vatinoxan | 0.4181 | 0.0995 | 90 | 4.204 | 0.0002 |
| BIMU8 - Vatinoxan | 0.39 | 0.1027 | 90.3 | 3.799 | 0.0008 |

Results are averaged over the levels of time

Degrees-of-freedom method: kenward-roger

P value adjustment: tukey method for comparing a family of 3 estimates

**Arterial partial pressure of carbon dioxide (**PaCO_2_)

Model: lmer (PaCO_2_ ~ treatment + time + (1|Sheep.ID))

Random effects:

| Groups | Name | Variance | Std.Dev. |
| --- | --- | --- | --- |
| Sheep.ID | (Intercept) | 11.38 | 3.373 |
| Residual |  | 19.91 | 4.462 |

Fixed effects:

|  | Estimate | Std.Error | df | t value | Pr(>\|t\|) |  |
| --- | --- | --- | --- | --- | --- | --- |
| (Intercept) | 41.6953 | 1.8438 | 12.8638 | 22.613 | <.0001 | *** |
| treatmentBIMU8 | -0.8011 | 1.1195 | 90.4147 | -0.716 | 0.4761 |  |
| treatmentVatinoxan | 1.7317 | 1.0689 | 90.0561 | 1.62 | 0.1087 |  |
| timepost etorphine | 10.2167 | 1.4873 | 89.9993 | 6.869 | <.0001 | *** |
| timepost naltrexone | 4.1824 | 1.5642 | 90.0641 | 2.674 | 0.0089 | ** |
| timepost treatment | 20.8906 | 1.2286 | 90.0576 | 17.004 | <.0001 | *** |

Signif. codes: 0 '***' 0.001 '**' 0.01 '*' 0.05 '.' 0.1 ' ' 1

Type III Analysis of Variance Table with Satterthwaite's method:

|  | Sum Sq | Mean Sq | NumDF | DenDF | F value | Pr(>F) |  |
| --- | --- | --- | --- | --- | --- | --- | --- |
| treatment | 112.5 | 56.27 | 2 | 90.255 | 2.8264 | 0.06448 | . |
| time | 7434.7 | 2478.24 | 3 | 90.05 | 124.4832 | <.0001 | *** |

Signif. codes: 0 '***' 0.001 '**' 0.01 '*' 0.05 '.' 0.1 ' ' 1

Pairwise comparison of time

| contrast | estimate | SE | df | t.ratio | p.value |
| --- | --- | --- | --- | --- | --- |
| baseline - post etorphine | -10.22 | 1.49 | 90 | -6.869 | <.0001 |
| baseline - post naltrexone | -4.18 | 1.56 | 90.1 | -2.673 | 0.0434 |
| baseline - post treatment | -20.89 | 1.23 | 90.1 | -17.002 | <.0001 |
| post etorphine - post naltrexone | 6.03 | 1.56 | 90.1 | 3.857 | 0.0012 |
| post etorphine - post treatment | -10.67 | 1.23 | 90.1 | -8.687 | <.0001 |
| post naltrexone - post treatment | -16.71 | 1.32 | 90 | -12.696 | <.0001 |

Results are averaged over the levels of treatment

Degrees-of-freedom method: kenward-roger

P value adjustment: Tukey method for comparing a family of 4 estimates

Pairwise comparison of treatment

| contrast | estimate | SE | df | t.ratio | p.value |
| --- | --- | --- | --- | --- | --- |
| Control - BIMU8 | 0.801 | 1.12 | 90.4 | 0.715 | 0.7554 |
| Control - Vatinoxan | -1.732 | 1.07 | 90.1 | -1.62 | 0.2426 |
| BIMU8 - Vatinoxan | -2.533 | 1.1 | 90.3 | -2.295 | 0.0616 |

Results are averaged over the levels of time

Degrees-of-freedom method: kenward-roger

P value adjustment: tukey method for comparing a family of 3 estimates

**Venous Admixture (Qs/Qt)**

Model: lmer (Qs/Qt ~ treatment + time + (1|Sheep.ID))

Random effects:

| Groups | Name | Variance | Std.Dev. |
| --- | --- | --- | --- |
| Sheep.ID | (Intercept) | 0.005188 | 0.07203 |
| Residual |  | 0.024892 | 0.15777 |

Fixed effects:

|  | Estimate | Std.Error | df | t value | Pr(>\|t\|) | signif |
| --- | --- | --- | --- | --- | --- | --- |
| (Intercept) | 0.11212 | 0.05238 | 27.13297 | 2.141 | 0.0414 | * |
| treatmentBIMU8 | -0.05725 | 0.03953 | 90.94051 | -1.448 | 0.1509 |  |
| treatmentVatinoxan | 0.07088 | 0.03779 | 90.15781 | 1.876 | 0.0639 | . |
| timepost etorphine | 0.50556 | 0.05259 | 90.02673 | 9.613 | <.0001 | *** |
| timepost naltrexone | 0.11572 | 0.0553 | 90.17688 | 2.093 | 0.0392 | * |
| timepost treatment | 0.63802 | 0.04343 | 90.15759 | 14.689 | <.0001 | *** |

Signif. codes: 0 '***' 0.001 '**' 0.01 '*' 0.05 '.' 0.1 ' ' 1

Type III Analysis of Variance Table with Satterthwaite's method:

|  | Sum Sq | Mean Sq | NumDF | DenDF | F value | Pr(>F) | signif |
| --- | --- | --- | --- | --- | --- | --- | --- |
| treatment | 0.2728 | 0.13641 | 2 | 90.595 | 5.4799 | 0.005668 | ** |
| time | 7.0599 | 2.3533 | 3 | 90.143 | 94.5385 | <.0001 | *** |

Signif. codes: 0 '***' 0.001 '**' 0.01 '*' 0.05 '.' 0.1 ' ' 1

Pairwise comparison of time

| contrast | estimate | SE | df | t.ratio | p.value |
| --- | --- | --- | --- | --- | --- |
| baseline - post etorphine | -0.506 | 0.0526 | 90 | -9.613 | <.0001 |
| baseline - post naltrexone | -0.116 | 0.0553 | 90.2 | -2.092 | 0.1636 |
| baseline - post treatment | -0.638 | 0.0434 | 90.1 | -14.685 | <.0001 |
| post etorphine - post naltrexone | 0.39 | 0.0553 | 90.2 | 7.047 | <.0001 |
| post etorphine - post treatment | -0.132 | 0.0434 | 90.1 | -3.049 | 0.0157 |
| post naltrexone - post treatment | -0.522 | 0.0465 | 90.1 | -11.224 | <.0001 |

Results are averaged over the levels of treatment

Degrees-of-freedom method: kenward-roger

P value adjustment: Tukey method for comparing a family of 4 estimates

Pairwise comparison of treatment

| contrast | estimate | SE | df | t.ratio | p.value |
| --- | --- | --- | --- | --- | --- |
| Control - BIMU8 | 0.0572 | 0.0396 | 90.9 | 1.445 | 0.3222 |
| Control - Vatinoxan | -0.0709 | 0.0378 | 90.1 | -1.875 | 0.1518 |
| BIMU8 - Vatinoxan | -0.1281 | 0.039 | 90.7 | -3.284 | 0.0041 |

Results are averaged over the levels of time

Degrees-of-freedom method: kenward-roger

P value adjustment: tukey method for comparing a family of 3 estimates

**Arterial partial pressure of oxygen (**PaO_2_)

Model: lmer (PaO_2_ ~ treatment + time + (1|Sheep.ID), data=data)

Random effects:

| Groups | Name | Variance | Std.Dev. |
| --- | --- | --- | --- |
| Sheep.ID | (Intercept) | 40.26 | 6.345 |
| Residual |  | 174.05 | 13.193 |

Fixed effects:

|  | Estimate | Std.Error | df | t value | Pr(>\|t\|) |  |
| --- | --- | --- | --- | --- | --- | --- |
| (Intercept) | 96.42 | 4.455 | 25.024 | 21.643 | <.0001 | *** |
| treatmentBIMU8 | 3.863 | 3.306 | 90.865 | 1.169 | 0.2456 |  |
| treatmentVatinoxan | -5.607 | 3.16 | 90.135 | -1.774 | 0.07936 | . |
| timepost etorphine | -46.65 | 4.398 | 90.014 | -10.608 | <.0001 | *** |
| timepost naltrexone | -14.48 | 4.624 | 90.153 | -3.131 | 0.00235 | ** |
| timepost treatment | -57.638 | 3.632 | 90.136 | -15.87 | <.0001 | *** |

Signif. codes: 0 '***' 0.001 '**' 0.01 '*' 0.05 '.' 0.1 ' ' 1

Type III Analysis of Variance Table with Satterthwaite's method:

|  | Sum Sq | Mean Sq | NumDF | DenDF | F value | Pr(>F) |  |
| --- | --- | --- | --- | --- | --- | --- | --- |
| treatment | 1508 | 754.2 | 2 | 90.543 | 4.3333 | 0.01595 | * |
| time | 54730 | 18243.3 | 3 | 90.122 | 104.8174 | <.0001 | *** |

Signif. codes: 0 '***' 0.001 '**' 0.01 '*' 0.05 '.' 0.1 ' ' 1

Pairwise comparison of time

| contrast | estimate | SE | df | t.ratio | p.value |
| --- | --- | --- | --- | --- | --- |
| baseline - post etorphine | 46.6 | 4.4 | 90 | 10.608 | <.0001 |
| baseline - post naltrexone | 14.5 | 4.63 | 90.1 | 3.13 | 0.0124 |
| baseline - post treatment | 57.6 | 3.63 | 90.1 | 15.865 | <.0001 |
| post etorphine - post naltrexone | -32.2 | 4.63 | 90.1 | -6.955 | <.0001 |
| post etorphine - post treatment | 11 | 3.63 | 90.1 | 3.025 | 0.0168 |
| post naltrexone - post treatment | 43.2 | 3.89 | 90.1 | 11.091 | <.0001 |

Results are averaged over the levels of treatment

Degrees-of-freedom method: kenward-roger

P value adjustment: Tukey method for comparing a family of 4 estimates

Pairwise comparison of treatment

| contrast | estimate | SE | df | t.ratio | p.value |
| --- | --- | --- | --- | --- | --- |
| Control - BIMU8 | -3.86 | 3.31 | 90.9 | -1.166 | 0.4763 |
| Control - Vatinoxan | 5.61 | 3.16 | 90.1 | 1.774 | 0.1842 |
| BIMU8 - Vatinoxan | 9.47 | 3.26 | 90.6 | 2.903 | 0.0128 |

Results are averaged over the levels of time

Degrees-of-freedom method: kenward-roger

P value adjustment: tukey method for comparing a family of 3 estimates

**Arterial oxygen content (CaO_2_)**

Model: lmer (CaO_2_~ treatment + time + (1|Sheep.ID))

Random effects:

| Groups | Name | Variance | Std.Dev. |
| --- | --- | --- | --- |
| Sheep.ID | (Intercept) | 0.1029 | 0.3208 |
| Residual |  | 0.3112 | 0.5579 |

Fixed effects:

|  | Estimate | Std.Error | df | t value | Pr(>\|t\|) | signif |
| --- | --- | --- | --- | --- | --- | --- |
| (Intercept) | 0.8851 | 0.2016 | 19.092 | 4.39 | 0.000312 | *** |
| treatmentBIMU8 | 0.1616 | 0.1399 | 90.6769 | 1.156 | 0.250911 |  |
| treatmentVatinoxan | -0.134 | 0.1336 | 90.1185 | -1.003 | 0.318776 |  |
| timepost etorphine | -0.9985 | 0.186 | 90.0278 | -5.369 | <.0001 | *** |
| timepost naltrexone | 0.3663 | 0.1956 | 90.1315 | 1.873 | 0.064271 | . |
| timepost treatment | -1.5587 | 0.1536 | 90.1197 | -10.148 | <.0001 | *** |

Signif. codes: 0 '***' 0.001 '**' 0.01 '*' 0.05 '.' 0.1 ' ' 1

Type III Analysis of Variance Table with Satterthwaite's method:

|  | Sum Sq | Mean Sq | NumDF | DenDF | F value | Pr(>F) | signif |
| --- | --- | --- | --- | --- | --- | --- | --- |
| treatment | 1.432 | 0.7159 | 2 | 90.429 | 2.3003 | 0.1061 |  |
| time | 60.373 | 20.1244 | 3 | 90.108 | 64.6603 | <.0001 | *** |

Signif. codes: 0 '***' 0.001 '**' 0.01 '*' 0.05 '.' 0.1 ' ' 1

Pairwise comparison of time

| contrast | estimate | SE | df | t.ratio | p.value |
| --- | --- | --- | --- | --- | --- |
| baseline - post etorphine | 0.998 | 0.186 | 90 | 5.369 | <.0001 |
| baseline - post naltrexone | -0.366 | 0.196 | 90.1 | -1.873 | 0.247 |
| baseline - post treatment | 1.559 | 0.154 | 90.1 | 10.146 | <.0001 |
| post etorphine - post naltrexone | -1.365 | 0.196 | 90.1 | -6.977 | <.0001 |
| post etorphine - post treatment | 0.56 | 0.154 | 90.1 | 3.646 | 0.0025 |
| post naltrexone - post treatment | 1.925 | 0.165 | 90.1 | 11.699 | <.0001 |

Results are averaged over the levels of treatment

Degrees-of-freedom method: kenward-roger

P value adjustment: Tukey method for comparing a family of 4 estimates

Pairwise comparison of treatment

| contrast | estimate | SE | df | t.ratio | p.value |
| --- | --- | --- | --- | --- | --- |
| Control - BIMU8 | -0.162 | 0.14 | 90.7 | -1.154 | 0.4838 |
| Control - Vatinoxan | 0.134 | 0.134 | 90.1 | 1.002 | 0.5773 |
| BIMU8 - Vatinoxan | 0.296 | 0.138 | 90.5 | 2.142 | 0.0871 |

Results are averaged over the levels of time

Degrees-of-freedom method: kenward-roger

P value adjustment: tukey method for comparing a family of 3 estimates

**Oxygen delivery (DO_2_)**

**Model: lmer (DO_2_~ treatment*time + (1|Sheep.ID), data=data)**

Random effects:

| Groups |  | Name | Variance | Std.Dev. |
| --- | --- | --- | --- | --- |
| Sheep.ID |  | (Intercept) | 5462 | 73.9 |
| Residual |  |  | 73506 | 271.1 |

Fixed effects:

|  | Estimate | Std. Error | df | t value | Pr(>\|t\|) | signif |
| --- | --- | --- | --- | --- | --- | --- |
| (Intercept) | 920.917 | 114.723 | 81.993 | 8.027 | 6.17E-12 | *** |
| treatmentBIMU8 | 29.633 | 156.531 | 84.262 | 0.189 | 0.850303 |  |
| treatmentVatinoxan | 86.917 | 156.531 | 84.262 | 0.555 | 0.580184 |  |
| timepost etorphine | -294.983 | 156.531 | 84.262 | -1.884 | 0.062945 | . |
| timepost naltrexone | 189.941 | 164.426 | 84.665 | 1.155 | 0.251266 |  |
| timepost treatment | -494.647 | 128.772 | 84.32 | -3.841 | 0.000236 | *** |
| treatmentBIMU8:timepost etorphine | -5.633 | 221.369 | 84.262 | -0.025 | 0.979758 |  |
| treatmentVatinoxan:timepost etorphine | -239.85 | 221.369 | 84.262 | -1.083 | 0.281684 |  |
| treatmentBIMU8:timepost naltrexone | 88.358 | 240.757 | 85.128 | 0.367 | 0.714528 |  |
| treatmentVatinoxan:timepost naltrexone | 593.775 | 227.02 | 84.475 | 2.616 | 0.01055 | * |
| treatmentBIMU8:timepost treatment | 40.322 | 183.941 | 84.707 | 0.219 | 0.827011 |  |
| treatmentVatinoxan:timepost treatment | -101.053 | 181.43 | 84.291 | -0.557 | 0.579015 |  |

Signif. codes: 0 '***' 0.001 '**' 0.01 '*' 0.05 '.' 0.1 ' ' 1

Type III Analysis of Variance Table with Satterthwaite's method:

|  | Sum sq | Mean Sq | NumDF | DenDF | F value | Pr(>F) |  |
| --- | --- | --- | --- | --- | --- | --- | --- |
| treatment | 318799 | 159399 | 2 | 84.945 | 2.1685 | 0.120637 |  |
| time | 11367173 | 3789058 | 3 | 84.497 | 51.5474 | < 2.2e-16 | *** |
| treatment:time | 1500788 | 250131 | 6 | 84.578 | 3.4029 | 0.004682 | ** |

Pairwise comparison of time across treatment:

*Treatment = control*

| Contrast | Estimate | SE | df | t.ratio | p.value |
| --- | --- | --- | --- | --- | --- |
| baseline – post etorphine | 295 | 157 | 84 | 1.884 | 0.2425 |
| baseline – post naltrexone | -189.9 | 165 | 84.4 | -1.154 | 0.6573 |
| baseline – post treatment | 494.6 | 129 | 84.1 | 3.841 | 0.0013 |
| post etorphine – post naltrexone | -484.9 | 165 | 84.4 | -2.946 | 0.0212 |
| post etorphine – post treatment | 199.7 | 129 | 84.1 | 1.55 | 0.4125 |
| post naltrexone – post treatment | 684.6 | 138 | 84.3 | 4.954 | <.0001 |

*Treatment = BIMU-8*

| Contrast | Estimate | SE | df | t.ratio | p.value |
| --- | --- | --- | --- | --- | --- |
| baseline – post etorphine | 300.6 | 157 | 84 | 1.92 | 0.2274 |
| baseline – post naltrexone | -278.3 | 176 | 84.9 | -1.581 | 0.3949 |
| baseline – post treatment | 454.3 | 132 | 84.8 | 3.454 | 0.0047 |
| post etorphine – post naltrexone | -578.9 | 176 | 84.9 | -3.289 | 0.0078 |
| post etorphine – post treatment | 153.7 | 132 | 84.8 | 1.169 | 0.6482 |
| post naltrexone – post treatment | 732.6 | 153 | 84.7 | 4.781 | <.0001 |

*Treatment = Vatinoxan*

| Contrast | Estimate | SE | df | t.ratio | p.value |
| --- | --- | --- | --- | --- | --- |
| baseline – post etorphine | 534.8 | 157 | 84 | 3.417 | 0.0053 |
| baseline – post naltrexone | -783.7 | 157 | 84 | -5.007 | <.0001 |
| baseline – post treatment | 595.7 | 128 | 84 | 4.661 | 0.0001 |
| post etorphine – post naltrexone | -1318.5 | 157 | 84 | -8.424 | <.0001 |
| post etorphine – post treatment | 60.9 | 128 | 84 | 0.476 | 0.9641 |
| post naltrexone – post treatment | 1379.4 | 128 | 84 | 10.793 | <.0001 |

Degrees-of-freedom method: kenward-roger

P value adjustment: tukey method for comparing a family of 4 estimates

Pairwise comparison of treatment across time:

*Baseline*

| Contrast | Estimate | SE | df | t.ratio | p.value |
| --- | --- | --- | --- | --- | --- |
| Control – BIMU-8 | -29.6 | 156.5 | 84 | -0.189 | 0.9804 |
| Control – Vatinoxan | -86.9 | 156.5 | 84 | -0.555 | 0.8441 |
| BIMU-8 – Vatinoxan | -57.3 | 156.5 | 84 | -0.366 | 0.9289 |

*Post etorphine*

| Contrast | Estimate | SE | df | t.ratio | p.value |
| --- | --- | --- | --- | --- | --- |
| Control – BIMU-8 | -24 | 156.5 | 84 | -0.153 | 0.9871 |
| Control – Vatinoxan | 152.9 | 156.5 | 84 | 0.977 | 0.5934 |
| BIMU-8 – Vatinoxan | 176.9 | 156.5 | 84 | 1.13 | 0.4982 |

*Post treatment*

| Contrast | Estimate | SE | df | t.ratio | p.value |
| --- | --- | --- | --- | --- | --- |
| Control – BIMU-8 | -70 | 97 | 85.6 | -0.721 | 0.7516 |
| Control – Vatinoxan | 14.1 | 91.8 | 84.1 | 0.154 | 0.987 |
| BIMU-8 – Vatinoxan | 84.1 | 95.6 | 85.4 | 0.88 | 0.6545 |

*Post naltrexone*

| Contrast | Estimate | SE | df | t.ratio | p.value |
| --- | --- | --- | --- | --- | --- |
| Control – BIMU-8 | -118 | 183.7 | 85.5 | -0.642 | 0.7971 |
| Control – Vatinoxan | -680.7 | 164.6 | 84.4 | -4.135 | 0.0002 |
| BIMU-8 – Vatinoxan | -562.7 | 176 | 84.9 | -3.197 | 0.0055 |

Degrees-of-freedom method: kenward-roger

P value adjustment: tukey method for comparing a family of 4 estimates

**Oxygen extraction ration (ER)**

Model: lmer (ER~ treatment + time + (1|Sheep.ID))

Random effects:

| Groups | Name | Variance | Std.Dev. |
| --- | --- | --- | --- |
| Sheep.ID | (Intercept) | 0.0003393 | 0.01842 |
| Residual |  | 0.0122094 | 0.1105 |

Fixed effects:

|  | Estimate | Std.Error | df | t value | Pr(>\|t\|) | signif |
| --- | --- | --- | --- | --- | --- | --- |
| (Intercept) | 0.4146012 | 0.0312531 | 72.034231 | 13.266 | <.0001 | *** |
| treatmentBIMU8 | 0.0401391 | 0.0275403 | 92.516454 | 1.457 | 0.148 |  |
| treatmentVatinoxan | -0.000609 | 0.026446 | 90.784462 | -0.023 | 0.982 |  |
| timepost etorphine | 0.0333333 | 0.0368321 | 90.43199 | 0.905 | 0.368 |  |
| timepost naltrexone | 0.0547467 | 0.0386947 | 90.841652 | 1.415 | 0.161 |  |
| timepost treatment | 0.131545 | 0.0303973 | 90.751674 | 4.328 | <.0001 | *** |

Signif. codes: 0 '***' 0.001 '**' 0.01 '*' 0.05 '.' 0.1 ' ' 1

Type III Analysis of Variance Table with Satterthwaite's method:

|  | Sum Sq | Mean Sq | NumDF | DenDF | F value | Pr(>F) | signif |
| --- | --- | --- | --- | --- | --- | --- | --- |
| treatment | 0.034841 | 0.01742 | 2 | 91.783 | 1.4268 | 0.2454 |  |
| time | 0.296204 | 0.098735 | 3 | 90.735 | 8.0867 | <.0001 | *** |

Signif. codes: 0 '***' 0.001 '**' 0.01 '*' 0.05 '.' 0.1 ' ' 1

Pairwise comparison of time

| contrast | estimate | SE | df | t.ratio | p.value |
| --- | --- | --- | --- | --- | --- |
| baseline - post etorphine | -0.0333 | 0.0368 | 90 | -0.905 | 0.8022 |
| baseline - post naltrexone | -0.0547 | 0.0387 | 90.5 | -1.413 | 0.4944 |
| baseline - post treatment | -0.1315 | 0.0304 | 90.4 | -4.324 | 0.0002 |
| post etorphine - post naltrexone | -0.0214 | 0.0387 | 90.5 | -0.553 | 0.9456 |
| post etorphine - post treatment | -0.0982 | 0.0304 | 90.4 | -3.229 | 0.0092 |
| post naltrexone - post treatment | -0.0768 | 0.0326 | 90.4 | -2.357 | 0.0931 |

Results are averaged over the levels of treatment

Degrees-of-freedom method: kenward-roger

P value adjustment: Tukey method for comparing a family of 4 estimates

Pairwise comparison of treatment

| contrast | estimate | SE | df | t.ratio | p.value |
| --- | --- | --- | --- | --- | --- |
| Control - BIMU8 | -0.040139 | 0.0277 | 92.3 | -1.45 | 0.32 |
| Control - Vatinoxan | 0.000609 | 0.0265 | 90.4 | 0.023 | 0.9997 |
| BIMU8 - Vatinoxan | 0.040748 | 0.0273 | 91.7 | 1.493 | 0.2988 |

Results are averaged over the levels of time

Degrees-of-freedom method: kenward-roger

P value adjustment: tukey method for comparing a family of 3 estimates
